# TCM‐Guided Targeted Therapies Against NLRP3 Inflammasome in NAFLD

**DOI:** 10.1002/iid3.70474

**Published:** 2026-06-17

**Authors:** Yanxia Zhang

**Affiliations:** ^1^ Shandong Xinzhonglu Traditional Chinese Medicine Hospital Jinan Shandong China

**Keywords:** NLRP3 inflammasome, non‐alcoholic fatty liver disease (NAFLD), precision medicine, therapeutic strategies, traditional Chinese medicine (TCM)

## Abstract

**Background:**

Non‐alcoholic fatty liver disease (NAFLD) is a prevalent metabolic disorder driven by inflammation, in which the NLRP3 inflammasome plays a central role. Current targeted inhibitors face challenges in patient stratification and long‐term safety.

**Methods:**

A narrative literature review was conducted using PubMed, Web of Science, China National Knowledge Infrastructure (CNKI), and Google Scholar from database inception to February 2026. Search terms included combinations of “non‐alcoholic fatty liver disease”, “NAFLD”, “NASH”, “NLRP3 inflammasome”, “traditional Chinese medicine”, “TCM”, “herbal medicine”, “syndrome differentiation”, and “precision medicine”. Peer‐reviewed original articles, systematic reviews, and clinical studies addressing NLRP3 in NAFLD or TCM‐based interventions targeting this pathway were prioritized.

**Results:**

TCM syndrome patterns (e.g., damp‐heat, phlegm‐damp) correlate with distinct NLRP3 activation states and inflammatory phenotypes. Herbal compounds such as berberine and curcumin, as well as classical TCM formulae, exert multi‐target effects by suppressing NLRP3 inflammasome assembly, enhancing antioxidant defenses, and modulating the gut‐liver axis. Based on this evidence, we propose an integrative “TCM Syndrome–NLRP3 Molecular Endotype–Precision Targeted Therapy” model that links specific TCM syndromes to canonical, non‐canonical, or sustained NLRP3 activation, thereby providing hypothesis‐driven strategies for personalized intervention.

**Discussion:**

This integrative framework bridges TCM holistic principles with modern inflammasome biology. It provides a theoretical basis for personalized, biomarker‐driven NAFLD therapies, highlighting the synergy between traditional medicine and precision hepatology.

## Graphical abstract

This graphical abstract illustrates TCM syndrome differentiation‐guided targeted strategies against NLRP3 inflammasome‐mediated non‐alcoholic fatty liver disease (NAFLD). Metabolic dysfunction and gut‐liver axis dysregulation drive NAFLD progression, activating the NLRP3 inflammasome (comprising NLRP3, ASC, caspase‐1) to trigger cytokine release (IL‐1β, IL‐18) and pyroptosis. Key TCM syndromes (e.g., dampness‐heat, phlegm obstruction) inform synergistic interventions: small‐molecule NLRP3 inhibitors, cytokine modulators, and TCM single herbs/formulae collectively disrupt inflammation cascades.

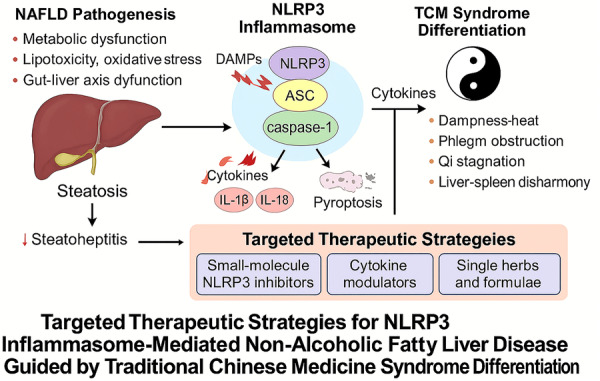

## 1 Introduction

Globally, non‐alcoholic fatty liver disease (NAFLD) is the most prevalent chronic liver disorder, affecting approximately 25% of the population and posing a significant healthcare burden due to its potential to progress from simple steatosis to non‐alcoholic steatohepatitis (NASH), cirrhosis, and hepatocellular carcinoma [1, 2]. The pathogenesis of NAFLD is highly complex and multifactorial, driven by genetic predisposition, insulin resistance, lipotoxicity, oxidative stress, gut microbiota dysbiosis, and chronic inflammation [3, 4]. Among these, inflammation, particularly that mediated by the innate immune system, is critical in the transition from steatosis to NASH and fibrosis. A central component in this inflammatory cascade is the NLRP3 inflammasome, a multiprotein cytosolic complex comprising NLRP3, the adapter protein ASC, and caspase‐1 [5]. Upon sensing metabolic danger signals, including free fatty acids (FFAs), cholesterol crystals, and reactive oxygen species (ROS), the NLRP3 inflammasome triggers caspase‐1, leading to the maturation and release of pro‐inflammatory cytokines IL‐1β and IL‐18 [6]. These pro‐inflammatory cytokines exacerbate hepatic inflammation, impair insulin signaling and induce pyroptotic cell death, forming a pathogenic feedback loop that accelerates NAFLD progression from steatosis to fibrosis and HCC [7].

Despite intensive research efforts, no pharmacological therapies have been approved specifically for NAFLD or NASH, largely due to disease heterogeneity and the lack of effective interventions targeting key inflammatory pathways [8]. The lack of approved therapies highlights the pressing need for novel, targeted, and personalized treatment strategies.

Traditional Chinese Medicine (TCM) offers a promising complementary, holistic approach to NAFLD management. Unlike single‐target pharmaceutical agents, TCM employs syndrome differentiation (Bian Zheng Lun Zhi), tailoring therapies to dynamic imbalances in Yin and Yang, Qi and Blood, and organ systems [9]. Emerging pharmacological studies have increasingly supported the idea that many TCM compounds exert therapeutic effects by modulating oxidative stress, metabolic signaling, and the NLRP3 inflammasome pathway [10, 11]. A growing body of evidence suggests that bioactive compounds from herbs such as Salvia miltiorrhiza, Curcuma longa, including berberine and baicalin, have shown potential to suppress NLRP3 inflammasome activation, thereby reducing hepatic inflammation, improving insulin sensitivity, and alleviating hepatocellular injury [12, 13]. Similarly, classical formulations, such as Huanglian Jiedu Decoction and Shenling Baizhu San, exhibit hepatoprotective effects consistent with modern insights into inflammation and metabolic regulation [14, 15].

This review aims to integrate current knowledge of NLRP3 inflammasome activation in NAFLD pathogenesis with the holistic framework of TCM. We propose a novel integrative model wherein specific TCM syndromes (e.g., damp‐heat and phlegm‐damp) are not merely descriptive clinical patterns but also reflect distinct molecular landscapes, particularly involving the activation state of the NLRP3 inflammasome and associated inflammatory cascades. By systematically bridging TCM syndrome differentiation with contemporary inflammasome biology, this review will explore how TCM diagnostic principles can guide patient stratification and how TCM‐derived therapies can precisely target NLRP3‐driven inflammation. This approach provides a coherent theoretical foundation for developing personalized, multi‐target therapeutic strategies for NAFLD.

For this narrative review, we conducted a comprehensive literature search using PubMed, Web of Science, China National Knowledge Infrastructure (CNKI), and Google Scholar databases. The search covered publications from database inception to February 2026. Keywords and MeSH terms included combinations of “non‐alcoholic fatty liver disease,” “NAFLD,” “NASH,” “NLRP3 inflammasome,” “traditional Chinese medicine,” “TCM,” “herbal medicine,” “phytotherapy,” “syndrome differentiation,” and “precision medicine.” We prioritized peer‐reviewed original research articles, systematic reviews, and clinical studies that investigated the role of the NLRP3 inflammasome in NAFLD or evaluated TCM‑based interventions targeting this pathway. Reference lists of relevant articles were also screened for additional studies. Although this review does not follow the PRISMA guidelines for systematic reviews, the described search strategy was employed to ensure a broad and balanced coverage of the available evidence.

## 2 Pathophysiology of NAFLD and Role of NLRP3 Inflammasome

### 2.1 NAFLD: From Metabolic Dysregulation to Cellular Stress

NAFLD encompasses a progressive spectrum from simple hepatic steatosis to NASH, advanced fibrosis, and ultimately cirrhosis or HCC. Simple steatosis, characterized by triglyceride accumulation in hepatocytes, is generally reversible [16]. However, in a subset of individuals, steatosis advances to NASH, marked by hepatocellular ballooning, lobular inflammation, and fibrogenesis [17].

The transition from steatosis to NASH is driven by multiple interrelated metabolic and stress pathways. Insulin resistance, a hallmark of obesity and type 2 diabetes mellitus, disrupts lipid homeostasis by enhancing adipose tissue lipolysis, increasing hepatic free fatty acid (FFA) influx, and stimulating de novo lipogenesis via upregulation of sterol regulatory element‑binding proteins (SREBPs) [18, 19, 20]. Hepatic lipid overload exceeds the capacities of lipid oxidation and export, resulting in ectopic lipid deposition and steatosis [21].

Notably, lipotoxic intermediates—including saturated fatty acids, ceramides, and diacylglycerol—induce endoplasmic reticulum (ER) stress, mitochondrial dysfunction, and excessive reactive oxygen species (ROS) generation [22]. Mitochondrial impairment causes defective β‑oxidation, electron leakage from the respiratory chain, and ROS overproduction. ROS drive lipid peroxidation, protein oxidative damage, and DNA injury, and trigger the release of mitochondrial DNA (mtDNA) into the cytoplasm. These cytosolic mtDNA fragments function as danger‑associated molecular patterns (DAMPs) that amplify sterile inflammation [23, 24].

The gut–liver axis constitutes an additional critical stress amplifier. Dysbiosis of intestinal microbiota compromises intestinal barrier integrity, leading to increased permeability (“leaky gut”) and translocation of microbial products such as lipopolysaccharide (LPS) and peptidoglycan into the portal vein. This activates hepatic Toll‑like receptors (TLRs) on Kupffer cells and hepatocytes, fueling pro‑inflammatory signaling cascades. Bacterial metabolites, including ethanol and secondary bile acids, further exacerbate hepatic injury [25, 26].

Ultimately, converging metabolic stressors—lipotoxicity, oxidative/ER stress, and gut‑derived endotoxins—collectively transform benign steatosis into a pro‑inflammatory and pro‑fibrotic hepatic microenvironment. These upstream insults serve as the primary triggers for subsequent innate immune activation, a process in which the NLRP3 inflammasome plays a pivotal intermediary role.

### 2.2 NLRP3 Inflammasome: A Central Hub Linking Metabolic Stress to Hepatic Inflammation and Fibrosis

#### 2.2.1 Structure and Activation Mechanisms

The NLRP3 inflammasome is a cytosolic multiprotein complex that functions as a critical pattern recognition receptor (PRR) of the innate immune system. It comprises three core components: the sensor NLRP3, the adapter ASC, and the effector caspase‑1 [27, 28].

Its activation typically follows a two‐step process. Signal 1 (priming), triggered by stimuli such as LPS or TNF‐α, upregulates the expression of NLRP3 and pro‐IL‐1β/pro‐IL‐18 via NF‐κB signaling. Subsequently, Signal 2 (activation) is initiated by diverse intracellular danger signals (e.g., ROS, K^+^ efflux, and lysosomal damage), leading to NLRP3 oligomerization, ASC speck formation, and caspase‐1 activation. Activated caspase‑1 then cleaves pro‑IL‑1β and pro‑IL‑18 into their mature, bioactive forms and processes gasdermin D (GSDMD) to execute pyroptosis, a pro‑inflammatory form of cell death [29]. In addition, a non‑canonical pathway involving caspase‑4/5 (humans) or caspase‑11 (mice) can be activated by intracellular LPS, further amplifying inflammasome activity via GSDMD cleavage [30, 31].

### 2.3 NLRP3 Activation in the NAFLD Microenvironment

In NAFLD, the metabolic and stress signals detailed in Section 2.1 directly supply the “Signal 2” required for NLRP3 inflammasome assembly. Mitochondrial dysfunction‑derived ROS and oxidized mtDNA are potent endogenous DAMPs that bind to and activate NLRP3 [30]. Lipotoxic lipids, such as ceramides and saturated fatty acids, trigger lysosomal destabilization and cathepsin B release, further promoting NLRP3 oligomerization. Collectively, these NAFLD‑specific danger signals converge on NLRP3, establishing it as a molecular rheostat that converts metabolic overload into an innate immune response.

### 2.4 Downstream Consequences: From Hepatocyte Injury to Systemic Insulin Resistance

Once activated, the NLRP3 inflammasome amplifies liver injury and drives NAFLD progression through four interconnected pathological axes. First, caspase‑1‑mediated GSDMD cleavage executes pyroptosis in hepatocytes, leading to cell swelling, membrane rupture, and release of intracellular DAMPs such as HMGB1 and ATP. This perpetuates a vicious cycle of sterile inflammation and recruits additional immune cells [6, 7]. Second, Kupffer cells—the liver‑resident macrophages—undergo M1 polarization upon inflammasome activation, secreting IL‑1β, IL‑18, TNF‑α, and chemokines that recruit bone marrow‑derived monocytes; these infiltrating macrophages amplify and sustain hepatic inflammation [5]. Third, IL‑1β and IL‑18 act as paracrine mediators that activate hepatic stellate cells (HSCs), with IL‑1β synergizing with TGF‑β to promote HSC transdifferentiation into collagen‑producing myofibroblasts, thereby driving extracellular matrix deposition and progressive fibrosis [7]. Fourth, IL‑1β impairs insulin signaling by mediating inhibitory serine phosphorylation of insulin receptor substrate‑1 (IRS‑1) in hepatocytes, adipose tissue, and skeletal muscle, reducing Akt pathway activity and glucose uptake. This fosters systemic insulin resistance, which in turn exacerbates hepatic lipogenesis and steatosis, closing a pathogenic feedback loop [18, 20].

### 2.5 The NLRP3 Inflammasome as a Therapeutic Hub

The NLRP3 inflammasome is therefore not merely one of many inflammatory mediators in NAFLD; it represents a central molecular hub that integrates diverse metabolic danger signals and transduces them into hepatocyte death, chronic inflammation, fibrotic remodeling, and metabolic dysregulation. This convergent position at the nexus of multiple pathogenic pathways renders it a uniquely attractive therapeutic target. Importantly, the diverse upstream activators (e.g., lipotoxicity, oxidative stress, and gut‐derived LPS) and downstream consequences (e.g., pyroptosis, fibrosis, and insulin resistance) of NLRP3 activation align closely with the multifaceted nature of TCM syndromes in NAFLD.

## 3 Targeted Therapeutic Strategies Against NLRP3 Inflammasome in NAFLD

Based on its central role as a molecular hub integrating metabolic stress with hepatic inflammation and fibrogenesis, the NLRP3 inflammasome has emerged as a mechanistically attractive therapeutic target in NAFLD. From an integrative medicine perspective, these emerging molecular targets and their modulators can be conceptually aligned with the therapeutic principles of TCM syndrome differentiation. This alignment is supported by a growing body of pharmacological evidence demonstrating that bioactive compounds from TCM herbs used to treat specific NAFLD syndromes (e.g., damp‐heat and phlegm‐damp) often exert their effects precisely through modulating pathways such as NLRP3 inflammasome signaling, AMPK activation, and antioxidant defenses [32, 33, 34]. This “herb‐compound‐target” evidence provides a rational basis for hypothesizing that modern targeted therapies (e.g., direct NLRP3 inhibitors and AMPK activators) may share convergent biological endpoints with the TCM goal of rectifying corresponding syndromes, although this integrative model awaits further clinical validation. Current strategies to inhibit the NLRP3 inflammasome in NAFLD include direct inflammasome inhibitors, upstream signal modulators, and blockade of downstream effectors. Although most candidates are in preclinical or early‐phase trials, they represent a growing arsenal of potential therapies to counteract inflammation‐driven liver injury.

### 3.1 Direct Inhibitors of the NLRP3 Inflammasome

Direct pharmacological targeting of the NLRP3 inflammasome constitutes one of the most rational and specific approaches to abrogate sterile hepatic inflammation. This strategy principally focuses on preventing inflammasome assembly, caspase‐1 activation, and pyroptotic signaling.

- MCC950 (CRID3): MCC950 is the most widely studied selective NLRP3 inhibitor. It binds to the NACHT domain of NLRP3, blocking its ATPase activity, which is essential for oligomerization and inflammasome assembly [35]. In preclinical models of NAFLD and NASH, MCC950 has consistently exhibited robust anti‐inflammatory effects, including suppression of IL‐1β secretion, inhibition of pyroptosis, attenuation of hepatic steatosis, and amelioration of fibrosis [5]. Notably, MCC950 displays high selectivity for NLRP3 over other inflammasomes such as AIM2 and NLRC4, supporting its potential as a precision anti‐inflammatory agent [36]. However, its clinical development has faced challenges. While a phase II trial in rheumatoid arthritis demonstrated potent anti‐inflammatory activity, further development in some indications was halted due to potential liver toxicity concerns observed in preclinical models, underscoring the need for rigorous safety evaluation in chronic liver diseases like NAFLD [35].
- CY‐09: CY‐09 is another ATP‐competitive inhibitor that directly targets the NLRP3 ATP‐binding motif. In high‐fat diet (HFD)‐induced models of NAFLD, CY‐09 treatment leads to reduced hepatic lipid deposition, suppressed inflammatory cytokine release, and enhanced histological features [37, 38]. Its favorable pharmacokinetics and oral bioavailability make it a promising drug candidate for translational development [39]. Beyond NAFLD, the therapeutic efficacy of CY‐09 has recently been extended to other inflammatory conditions, including acute lung injury, myocardial infarction‐induced ventricular remodeling, and inner ear hemorrhage, further supporting its broad anti‐inflammatory potential [40, 41, 42].
- OLT1177 (Dapansutrile): OLT1177, an orally bioavailable β‐sulfonyl nitrile compound, has demonstrated potent NLRP3 inhibition in various inflammatory diseases. Although initially developed for gout and osteoarthritis, its demonstrated ability to reduce hepatic inflammation and metabolic derangement in preclinical NAFLD models indicates broader therapeutic applications [43, 44]. Human trials in inflammatory conditions have documented an acceptable safety profile, facilitating future investigation in NAFLD populations [45].

### 3.2 Inhibition of Upstream Activators

Complementary to directly blocking the inflammasome complex, modulating upstream metabolic and oxidative stressors offers a more integrative approach to attenuate NLRP3 activation and its downstream consequences.

- ROS Scavenging: Excessive ROS, generated via mitochondrial dysfunction or lipotoxicity, is a principal activator of the NLRP3 inflammasome in hepatocytes and immune cells. Antioxidants such as N‐acetylcysteine (NAC), mito‐TEMPO, and resveratrol have demonstrated efficacy in NAFLD models by neutralizing ROS and reducing lipid peroxidation [46, 47, 48]. These compounds not only prevent NLRP3 activation but also augment mitochondrial function and diminish hepatic injury.
- Thioredoxin‐Interacting Protein (TXNIP): TXNIP is a redox‐sensitive protein that dissociates from thioredoxin under oxidative conditions and binds to NLRP3, promoting inflammasome assembly [49]. Bioactive flavonoids exemplified by quercetin and curcumin inhibit this interaction, thereby suppressing inflammasome activation. These compounds confer dual antioxidant and anti‐inflammatory properties, rendering them attractive for multifactorial diseases like NAFLD [49].
- AMPK Activation: AMP‐activated protein kinase (AMPK) is a central metabolic sensor that exerts anti‐inflammatory effects, in part mediated by inhibiting NLRP3 inflammasome activation. Pharmacologic activators such as metformin, berberine, and AICAR not only restore energy homeostasis but also downregulate NF‐κB and TXNIP signaling pathways, thereby indirectly mitigating inflammasome activity [50, 51, 52]. These agents have demonstrated improvements in hepatic lipid profiles, insulin sensitivity, and inflammation, reinforcing their role as metabolic‐immunologic modulators.

### 3.3 Inhibition of Downstream Effectors

Targeting the downstream effectors of the NLRP3 inflammasome provides a strategy to attenuate its pathological consequences while circumventing direct interference with core assembly processes.

- IL‐1β Blockade: IL‐1β is a major effector cytokine released by the NLRP3 inflammasome, implicated in hepatic inflammation, fibrosis, and systemic insulin resistance. Anakinra, a recombinant IL‐1 receptor antagonist, and canakinumab, a monoclonal antibody against IL‐1β, have undergone evaluation in metabolic and cardiovascular diseases. Although dedicated clinical trials in NAFLD are lacking, evidence from related disease models and mechanistic studies supports their potential in mitigating hepatic inflammation and preventing fibrosis progression [53].
- Caspase‐1 Inhibition: Caspase‐1 catalyzes the proteolytic maturation of IL‐1β, IL‐18, and execution of pyroptosis via gasdermin D cleavage [54]. VX‐765, an oral caspase‐1 inhibitor, has shown promising results in reducing hepatic cytokine levels, alleviating steatohepatitis, and improving insulin sensitivity in murine models. Its development in chronic inflammatory diseases may extend to NAFLD pending safety validation [54].
- Gasdermin D Modulation: The therapeutic potential of targeting GSDMD‐mediated pyroptosis in NASH is strongly supported by genetic evidence (Gsdmd^−^/^−^ mice show attenuated steatohepatitis) and pharmacological evidence (multiple natural products and herbal formulas reduce GSDMD cleavage concomitant with improved histopathology) [55, 56, 57]. However, specific small‐molecule inhibitors of GSDMD cleavage have not yet been evaluated in NASH models.

### 3.4 Challenges and Limitations

Despite encouraging advances, the clinical translation of NLRP3‐targeted therapies in NAFLD faces several interconnected challenges that must be addressed [58]. First, while direct inhibitors such as MCC950 demonstrate high selectivity, concerns persist regarding potential off‐target effects and the long‐term implications of suppressing a key component of innate immunity [36, 59]. Given the physiological role of NLRP3 in host defense, chronic inhibition necessitates a careful risk‐benefit assessment for a lifelong condition like NAFLD. Second, achieving effective and liver‐specific drug delivery remains a major pharmacological hurdle. Ensuring sufficient therapeutic concentration at the hepatic site of action while minimizing systemic exposure is crucial for both efficacy and safety, yet current delivery strategies often lack the required precision, underscoring the need for advanced liver‐tropic delivery systems. Third, the marked etiological and phenotypic heterogeneity of NAFLD itself complicates therapeutic development. A uniform strategy targeting NLRP3 is unlikely to succeed across all patient subtypes, highlighting the imperative for effective stratification—potentially guided by TCM syndrome differentiation or molecular endotyping—to identify the subgroups most likely to respond [60]. Finally, progress in this field is significantly hampered by the absence of validated, non‐invasive biomarkers capable of reliably quantifying intrahepatic NLRP3 activity. Such biomarkers are a critical prerequisite not only for enriching clinical trial populations but also for objectively monitoring treatment response [60], thereby forming a foundational element for future precision therapy in NAFLD.

Moreover, the available evidence on NLRP3 inhibitors is not entirely consistent. For instance, while MCC950 has shown robust efficacy in multiple preclinical NASH models, its clinical development has been hindered by liver toxicity signals in some preclinical species [61], raising questions about the translatability of its therapeutic window. Conversely, OLT1177 has demonstrated a favorable safety profile in phase I/II trials for other inflammatory conditions [62], yet its efficacy in NAFLD remains to be established. Such discrepancies underscore the need for comparative head‐to‐head studies and a deeper understanding of context‐dependent NLRP3 functions. Furthermore, most preclinical studies have utilized young, male, high‐fat diet‐fed rodents, which may not fully recapitulate the heterogeneity of human NAFLD, potentially contributing to the inconsistency between preclinical promise and clinical setbacks.

### 3.5 Translational Outlook

The emergence of NLRP3 inflammasome‐targeted therapies has opened new therapeutic horizons for inflammation‐driven liver diseases such as NAFLD. Integration of NLRP3‐targeted therapies with metabolic modulators may offer synergistic benefits. Notably, preclinical studies in obesity models have demonstrated that combining NLRP3 inhibitors (e.g., NT‐0796 and VTX3232) with GLP‐1 receptor agonists (e.g., semaglutide) yields additive metabolic improvements, including enhanced weight loss and reduced hepatic steatosis [63, 64]. While PPAR agonists (δ/γ) have been shown to suppress NLRP3 inflammasome activation and ameliorate NAFLD in monotherapy settings [65], their combination with direct NLRP3 inhibitors remains unexplored. Similarly, multiple TCM herbal formulas (e.g., Zexie–Baizhu and Huanglian–Hongqu) exert anti‐NAFLD effects via NLRP3 pathway inhibition as single agents [66, 67], yet whether they confer additional benefit when combined with synthetic NLRP3 inhibitors warrants further investigation. As research continues to unravel the complex crosstalk between metabolism and innate immunity, precision‐targeted interventions for NAFLD focused on NLRP3 modulation are anticipated to evolve into a fundamental cornerstone of future therapeutic strategies [3].

## 4 TCM Perspective on NAFLD

Although NAFLD is not explicitly delineated as a standalone disease entity in classical TCM literature, its clinical manifestations, such as fatigue, hypochondriac discomfort, abdominal distension, and greasy tongue coating, correspond to several traditional syndromes, including “liver qi stagnation,” “phlegm‐damp retention,” “damp‐heat accumulation,” and “blood stasis” [68]. TCM theorizes NAFLD pathogenesis as arising from dysregulation of the liver–spleen axis, modulated by exogenous factors (improper diet and sedentary lifestyle) and endogenous imbalances (emotional stress and constitutional deficiency). These pathophysiological imbalances initiate a cascade of pathological patterns, providing a basis for individualized diagnosis and treatment according to the principle of syndrome differentiation (Bian Zheng Lun Zhi).

### 4.1 TCM Syndromes and NAFLD Pathogenesis

From the TCM perspective, NAFLD is primarily characterized by impaired liver function in maintaining smooth qi flow and compromised spleen capacity to transform and transport dampness. This dual dysfunction leads to the generation of phlegm and blood stasis, which obstruct the liver channels and contribute to lipid accumulation and inflammatory damage in the liver [69].

- Liver qi stagnation: Emotional stress disrupts liver qi circulation, impairing the regulation of blood and bile flow. This stagnation may progress to heat, contributing to inflammatory changes [70].
- Spleen deficiency and dampness: A deficient spleen is incapable of metabolizing fluids, leading to internal dampness, which may coalesce into phlegm and contribute to hepatic steatosis [71].
- Phlegm‐damp retention: Accumulated dampness and phlegm obstruct qi flow and hinder the liver's dispersing function, further exacerbating lipid deposition [70].
- Damp‐heat accumulation: Retained dampness transmutes into heat, provoking inflammatory processes that align with histological features of NASH [72].
- Qi stagnation with blood stasis: Chronic stagnation impairs blood circulation, leading to hepatic microcirculatory dysfunction and fibrogenesis [71].
- Liver–kidney yin deficiency: In advanced or chronic cases, depletion of liver and kidney yin may impair tissue repair and detoxification, further aggravating hepatic injury [73].

These syndromes often coexist or dynamically transform, reflective of the dynamic and systemic progression of NAFLD within TCM theory.

### 4.2 TCM Therapeutic Strategies

Accordingly, the therapeutic strategies in TCM are tailored to address these specific syndromes and their underlying molecular mechanisms:

- Phlegm‐damp syndrome is associated with impaired lipid metabolism, involving dysregulation of peroxisome proliferator‐activated receptors (PPARs) and AMPK pathways, which are critical in lipid oxidation and lipogenesis [20].
- Damp‐heat syndrome shows increased hepatic inflammation, with elevated levels of IL‐1β, IL‐18, and enhanced activation of the NLRP3 inflammasome, suggesting a linkage between this TCM pattern and inflammasome‐mediated immune responses [5].
- Qi stagnation and blood stasis reflect vascular dysfunction, hypoxia, and fibrogenic activation, paralleling upregulation of TGF‐β, PDGF, and other fibrosis‐related signaling cascades [74].
- Liver qi stagnation also correlates with systemic stress responses, including activation of the hypothalamic–pituitary–adrenal (HPA) axis and oxidative stress pathways, promoting mitochondrial damage and ROS generation, thereby indirectly enhancing NLRP3 activation [75].

This growing body of evidence provides a molecular rationale for TCM diagnosis and treatment in NAFLD, bridging the conceptual gap between ancient theory and modern biomedicine. It also underscores the relevance of TCM syndrome patterns in identifying NAFLD subtypes with distinct inflammatory and metabolic profiles [69, 76].

### 4.3 Integration With Precision Medicine

The individualized and holistic framework of TCM aligns closely with the goals of precision medicine, which seeks to tailor treatments based on genetic, biomarker, phenotypic, and psychosocial characteristics. TCM's syndrome differentiation offers an established conceptual stratification framework that is increasingly being integrated with molecular phenotyping in NAFLD [77, 78]. Based on a comprehensive synthesis of current pharmacological and mechanistic evidence, we herein propose a novel three‐dimensional integrative model of “TCM Syndrome‐NLRP3 Molecular Endotype‐Precision Targeted Therapy” for NAFLD. To our knowledge, this is the first systematic attempt to map core TCM syndromes of NAFLD to distinct NLRP3 activation modes (canonical, non‑canonical, and sustained canonical activation) and to define corresponding hypothesis‑driven targeted intervention strategies. We emphasize that this model represents a theoretical framework that requires rigorous prospective validation.

The model establishes a one‑to‑one correlation between NAFLD TCM syndromes and NLRP3 molecular endotypes, as hypothesized below:

- Phlegm‐damp retention syndrome, which is clinically characterized by prominent disturbances in lipid metabolism [76], is hypothesized to be tightly linked to sustained canonical NLRP3 activation driven by metabolic stress (e.g., lipotoxicity and ceramides). CY‑09, a direct NLRP3 ATPase inhibitor, reduces hepatic steatosis and inflammasome assembly in NAFLD models [38], while berberine suppresses NLRP3 through AMPK‑dependent pathways [79]. These complementary mechanisms suggest that patients with this endotype might derive particular benefit from combining a lipid‑metabolism modulator with an NLRP3 ATPase inhibitor (e.g., berberine plus CY‑09)—a combinatorial hypothesis that awaits experimental testing.
- Damp‐heat accumulation syndrome is associated with elevated systemic inflammatory markers and an increased risk of fibrosis [76]. The syndrome is proposed to reflect synergistic activation of canonical and non‑canonical NLRP3 pathways, triggered by oxidative stress and gut‑derived LPS. Curcumin inhibits NLRP3 inflammasome activation via multiple mechanisms [80], and MCC950 is a prototypical NLRP3 inhibitor with proven efficacy in NASH models, albeit accompanied by safety concerns. It is therefore plausible that NLRP3 pan‑inhibitors combined with ROS scavengers (e.g., curcumin plus MCC950) could represent an optimal intervention for this inflammatory endotype; this combination has not, to our knowledge, been experimentally evaluated.
- Qi stagnation with blood stasis syndrome correlates with advanced fibrosis in NAFLD. Mechanistically, hepatocyte pyroptosis releases NLRP3 inflammasome particles that activate hepatic stellate cells (HSCs) in a paracrine manner. Salvia miltiorrhiza extracts have been shown to inhibit HSC activation and attenuate liver fibrosis in preclinical models [74]. Consequently, anti‑fibrotic therapies combined with downstream inflammasome effector blockers (e.g., Salvia miltiorrhiza extracts plus IL‑1β antagonists) may prove more effective in this subgroup—a hypothesis that warrants dedicated preclinical investigation.
- Liver qi stagnation syndrome represents a milder, stress‑associated phenotype often modeled by chronic restraint stress in rodents [75]. In this context, NLRP3 may be primed without proceeding to full inflammasome assembly, possibly driven by neuroendocrine stress mediators. Xiaoyao Powder, a classic formula indicated for this syndrome, has been reported to ameliorate liver injury and metabolic disturbances in experimental models of liver depression [81]. Thus, mild anti‑inflammatory interventions (e.g., Xiaoyao Powder) may be sufficient to reverse the pathological state in this subgroup, although direct evidence linking this syndrome to incomplete NLRP3 activation remains sparse.

Integrating TCM syndrome diagnosis with modern biomarkers (NLRP3 activation status, cytokine profiles, and lipidomic signatures) based on this three‑dimensional model could facilitate more refined treatment algorithms, accelerating the development of a unique model of integrative precision hepatology. This model not only provides a mechanistic rationale for TCM syndrome differentiation but also offers a practical, hypothesis‑driven guide for clinical stratification of NAFLD patients receiving NLRP3‑targeted therapies.

## 5 TCM‐Based Interventions Targeting NLRP3: Herbal Compounds and Classical Formulae

**FIGURE 1:**
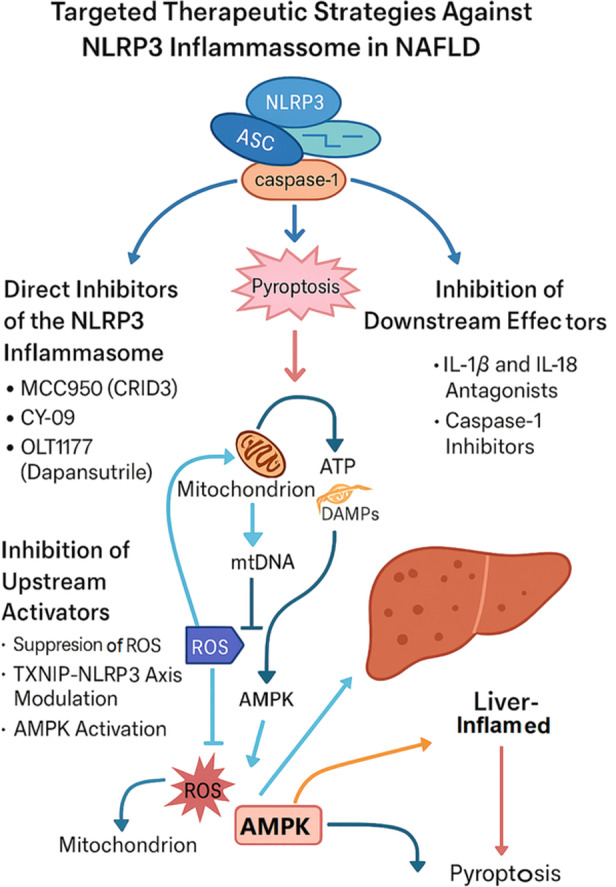
Targeted therapeutic strategies against the NLRP3 inflammasome in NAFLD. This figure summarizes three major classes of therapeutic interventions targeting the NLRP3 inflammasome pathway: direct NLRP3 inhibitors (MCC950, CY‐09, and OLT1177) block inflammasome assembly by binding to the NACHT domain and inhibiting ATPase activity; upstream activator modulators suppress NLRP3 activation by scavenging ROS, disrupting the TXNIP–NLRP3 interaction, or activating AMPK to restore metabolic homeostasis; and downstream effector blockers target IL‑1β/IL‑18 signaling or caspase‑1 to attenuate inflammation and pyroptosis. Key cellular events—including mitochondrial damage (mtDNA and ROS release), ATP‑triggered danger signaling, and pyroptotic cell death—are also depicted as central triggers or consequences of NLRP3 activation. Abbreviations: AMPK, AMP‑activated protein kinase; ATP, adenosine triphosphate; DAMP, damage‑associated molecular pattern; IL, interleukin; mtDNA, mitochondrial DNA; NAFLD, non‑alcoholic fatty liver disease; NLRP3, nucleotide‑binding oligomerization domain‑like receptor family pyrin domain‑containing 3; ROS, reactive oxygen species; TXNIP, thioredoxin‑interacting protein.

**FIGURE 2:**
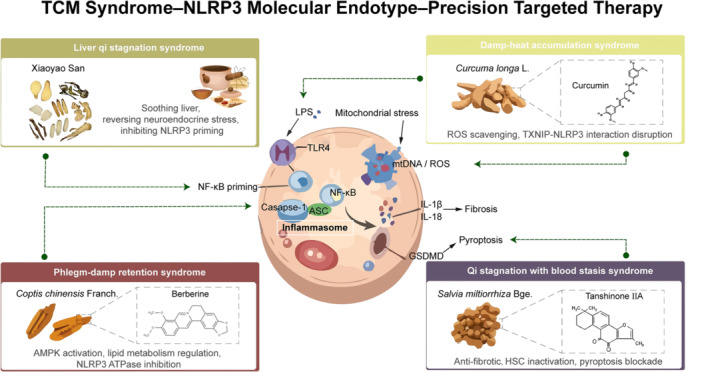
Correlation between TCM syndrome differentiation of NAFLD and NLRP3 molecular targets. This schematic summarizes the core TCM syndromes of NAFLD (phlegm‐damp retention, damp‐heat accumulation, qi stagnation with blood stasis, and liver qi stagnation) and their corresponding NLRP3 activation modes, as well as the key molecular pathways/mediators involved in each syndrome. It clarifies the molecular basis of TCM syndromes centered on NLRP3 inflammasome, providing a visual framework for syndrome‐guided precision targeting of NLRP3‐mediated hepatic inflammation in NAFLD.

TCM offers a long‐standing tradition for managing liver disorders, featuring a rich pharmacopeia composed of multi‐component herbal medicines capable of modulating complex pathological networks. Recent pharmacological studies have increasingly focused on the ability of TCM‐derived compounds and classical formulas to inhibit NLRP3 inflammasome activation, thereby attenuating hepatic inflammation and fibrosis in NAFLD. This section systematically reviews key herbal ingredients and formulae with Figure 1 and Figure 2 demonstrated anti‐NLRP3 efficacy and underlying molecular mechanisms.

### 5.1 Isolated Herbal Compounds Targeting NLRP3 Inflammasome

A variety of bioactive compounds purified from TCM herbs have shown direct or indirect inhibitory effects on the NLRP3 inflammasome and its downstream signaling pathways.

- Curcumin (from Curcuma longa): As a classic TCM herb with clearing heat and eliminating dampness effect, curcumin is the core therapeutic component for TCM Damp‐Heat Accumulation Syndrome. Mechanistically, curcumin suppresses NLRP3 inflammasome activation by inhibiting NF‐κB signaling, reducing ROS production, and disrupting the TXNIP–NLRP3 interaction. In NAFLD models, curcumin administration reduces IL‐1β secretion, hepatic steatosis, and fibrotic markers, highlighting one mechanism underlying its therapeutic potential [11, 80].
- Quercetin (found in many TCM herbs such as Sophora japonica): Quercetin attenuates NLRP3 activation by scavenging ROS and inhibiting mitochondrial dysfunction [82]. It also modulates AMPK/SIRT1 signaling to restore metabolic homeostasis [83]. Experimental studies report its efficacy in reducing liver inflammation and fibrosis in diet‐induced NAFLD models [84].
- Berberine (from Coptis chinensis): In TCM practice, berberine is a first‐line therapeutic component for Phlegm‐Damp Retention Syndrome, and it is a typical herb endowed with the effects of invigorating the spleen and resolving phlegm. Berberine activates AMPK, suppresses NF‐κB, and downregulates NLRP3 expression. Berberine administration is associated with improved gut barrier function and microbiota composition, potentially contributing to reduced endotoxin‐mediated inflammasome priming. Berberine improves insulin sensitivity and lipid metabolism, offering a multifaceted approach to NAFLD management [52, 79].
- Andrographolide (from Andrographis paniculata): This diterpenoid lactone inhibits NLRP3 inflammasome assembly by interfering with the ROS/TXNIP axis and attenuates inflammatory cytokine release. Its hepatoprotective effects have been demonstrated in various liver injury models [58, 85].
- Ginsenosides (from Panax ginseng): Several ginsenosides inhibit NLRP3‐mediated pyroptosis by suppressing mitochondrial ROS and promoting mitophagy, thus preventing inflammasome activation. They also regulate lipid metabolism and oxidative stress, showing benefits in metabolic liver diseases [9, 86].

### 5.2 Classical TCM Formulae Modulating NLRP3 Inflammasome

Numerous well‐known TCM prescriptions have been validated to exert anti‐inflammatory and anti‐fibrotic effects partially mediated via modulation of the NLRP3 inflammasome pathway.

- Liuwei Dihuang Decoction: Traditionally prescribed for liver–kidney yin deficiency, this formula has demonstrated downregulation of NLRP3, caspase‐1, and IL‐1β expression in experimental NAFLD. Its multi‐herbal composition confers synergistic benefits by mitigating oxidative stress and restoring mitochondrial function [87].
- Shaoyao‐Gancao Decoction: Known for regulating qi and blood, this formula reduces hepatic inflammation and fibrosis by suppressing NF‐κB signaling and inhibiting NLRP3 activation in liver macrophages [88].
- Danggui Shaoyao San: This classic formula improves microcirculation and alleviates blood stasis, thereby indirectly mitigating inflammasome activation linked to fibrotic progression [89].
- Huanglian Jiedu Decoction: Traditionally used to clear heat and dry dampness, it has been shown to reduce hepatic IL‐1β and NLRP3 levels, suggesting a direct anti‐inflammasome effect [14].
- Yinchenhao Decoction: Employed for damp‐heat syndromes of the liver, this formula modulates the gut–liver axis, reduces endotoxemia, and suppresses NLRP3 inflammasome‐mediated inflammation [90].

### 5.3 Mechanistic Synergy of Multi‐Component TCM Therapies

The complexity of TCM formulations allows simultaneous modulation of multiple NAFLD pathological axes:

- Anti‐oxidative effects: Many TCM herbs reduce mitochondrial ROS, a key NLRP3 activator [22].
- Anti‐inflammatory pathways: Suppression of NF‐κB and MAPK signaling attenuates inflammasome priming [10].
- Metabolic regulation: AMPK/SIRT1 activation restores lipid and glucose homeostasis [52].
- Gut microbiota modulation: Strengthening intestinal barrier integrity reduces LPS‐induced inflammasome priming [15].
- Anti‐fibrotic actions: Inhibition of TGF‐β and extracellular matrix deposition prevents disease progression [74].

This multi‐targeted approach aligns with the holistic philosophy of TCM, potentially offering superior efficacy and fewer side effects compared to single‐target drugs [77].

### 5.4 Challenges and Future Directions

Despite promising preclinical evidence supporting the anti‐NLRP3 efficacy of TCM‐derived compounds and classical formulas, the translation to clinical practice requires bridging a significant evidence gap. Although some randomized controlled trials (RCTs) have shown beneficial effects of TCM formulations on liver enzymes and ultrasound features in NAFLD patients [91, 92], large‐scale, multi‐center RCTs with histological endpoints and mechanistic correlates (e.g., NLRP3 activity) are still lacking. Several critical challenges impede their clinical translation and widespread application for NAFLD treatment, which need to be addressed with innovative strategies.

First, the active component content of herbal raw materials varies significantly depending on growing regions, harvesting times, and processing methods, leading to poor consistency and reproducibility of TCM preparations. Therefore, it is essential to establish unified quality control standards and fingerprinting techniques for both herbal materials and their formulations [87]. In addition, most core bioactive compounds (e.g., curcumin, berberine, and andrographolide) exhibit poor water solubility and low oral bioavailability, with insufficient intestinal absorption resulting in plasma concentrations below the therapeutic window. To address this, novel formulation strategies such as nanosuspensions, lipid‐based delivery systems, and prodrugs are required to improve their solubility and achieve targeted delivery to the liver [93]. Meanwhile, classical TCM formulas exert therapeutic effects through synergistic interactions of multiple active components acting on various molecular targets. Dissecting the individual contribution of each component and identifying the key active moieties responsible for NLRP3 inhibition demand integrated approaches combining systems pharmacology, network pharmacology, and experimental validation [94]. Finally, most of the current evidence supporting TCM‐based therapies for NAFLD stems from preclinical studies and small‐scale observational trials. Well‐designed, large‐sample randomized controlled clinical trials with long‐term follow‐up are urgently needed to validate the efficacy and safety of TCM interventions in NLRP3‐mediated NAFLD, as well as to identify optimal treatment regimens and suitable patient populations [87].

Integration of modern molecular techniques with traditional knowledge is essential to optimize TCM interventions targeting the NLRP3 inflammasome and advance precision hepatology [77]. Addressing regulatory heterogeneity and developing evidence‑based integration pathways—such as syndrome‑stratified trials and joint specialist collaboration—will be essential to position TCM as a credible component of precision NAFLD management.

## 6 Summary and Future Perspectives

### 6.1 Summary

NAFLD epitomizes a multifaceted metabolic‐inflammatory disorder with increasing global prevalence and significant health burden. The nucleotide‐binding oligomerization domain‐like receptor family pyrin domain‐containing 3 (NLRP3) inflammasome has emerged as a pivotal contributor to the initiation and progression of NAFLD by orchestrating hepatic inflammation, pyroptotic cell death, and fibrosis. Targeting the NLRP3 inflammasome thus offers a promising therapeutic avenue to intercept disease advancement at the inflammatory nexus.

This review systematically delineates the pathophysiological role of NLRP3 inflammasome in NAFLD, emphasizing its activation mechanisms by metabolic stressors, mitochondrial dysfunction, and gut‐derived endotoxins. We comprehensively summarize current targeted interventions, ranging from direct NLRP3 inhibitors and upstream modulators (e.g., antioxidants and AMPK activators) to downstream effectors such as IL‐1β antagonists and caspase‐1 inhibitors, highlighting their therapeutic potential and translational challenges.

A distinctive and novel contribution of this review is the establishment of a bidirectional bridge between TCM syndrome differentiation and NLRP3 inflammasome biology, culminating in the first proposal of the “TCM Syndrome–NLRP3 Molecular Endotype–Precision Targeted Therapy” three‑dimensional integrative model for NAFLD. This model systematically maps core TCM syndromes to specific NLRP3 activation states (canonical, non‑canonical, and sustained canonical) and generates four testable hypotheses linking diagnostic patterns to discrete molecular mechanisms and corresponding targeted interventions. We demonstrate that: (i) TCM patterns are not merely descriptive categories but can be re‑interpreted as quantifiable NLRP3‑centered molecular endotypes; (ii) syndrome‑guided selection of herbal and combination therapies can be rationalized through this model, enabling precision targeting of the inflammasome pathway; and (iii) this integrative framework transforms TCM's holistic individualization into a mechanism‑informed stratification system for precision hepatology. Moreover, we critically evaluate conflicting preclinical and clinical data on NLRP3‑targeted therapies and TCM interventions, identifying key knowledge gaps and sources of discrepancy. By consistently linking diagnostic patterns to molecular drivers and therapeutic actions throughout Sections 4 and 5, we provide a novel, reproducible integrative paradigm for TCM‑guided targeted therapy of NLRP3‑mediated NAFLD. We hope this model will serve as a methodological foundation and hypothesis‑generating platform for future integrative precision hepatology research.

### 6.2 Future Research Directions

- Elucidating Molecular Mechanism: Further dissection of TCM syndrome‐specific molecular signatures and their correlation with inflammasome activation is warranted. Omics technologies combined with systems biology can unravel the complex multi‐component interactions underlying TCM therapies.
- Advancing Biomarker Development: Identification of reliable, non‐invasive biomarkers reflecting NLRP3 inflammasome activity and TCM syndrome states will enable patient stratification, treatment monitoring, and personalized therapeutic regimens.
- Enhancing Pharmacological Optimization: Improving bioavailability and targeting of herbal compounds through novel drug delivery systems (e.g., nanoparticles and liposomes) will enhance efficacy and reduce off‐target effects.
- Conducting Clinical Trials: Well‐designed, large‐scale randomized controlled trials integrating TCM interventions with standard care are crucial to validate safety, efficacy, and mechanistic effects in diverse NAFLD populations.
- Fostering Precision Medicine Integration: Combining TCM syndrome differentiation with genomic, metabolomic, and immunologic profiling holds promise for the development of precision hepatology models that optimize NLRP3‐targeted treatments tailored to individual patient pathophenotypes.
- Regulatory science and implementation research: Developing fit‑for‑purpose regulatory frameworks and pragmatic integration models to accelerate the translation of TCM‑guided NLRP3‑targeted therapies into mainstream hepatology practice.

## 7 Concluding Remarks

Harnessing the synergy between TCM and contemporary biomedical research possesses the potential to transform NAFLD management through the provision of personalized, multi‐dimensional therapeutic strategies targeting the NLRP3 inflammasome. This integrative approach aligns ancient wisdom with cutting‐edge science, potentially improving clinical outcomes and mitigating the escalating global NAFLD burden.

## Author Contributions

**Yanxia Zhang:** conceptualization, investigation, writing – original draft, methodology, validation, visualization, writing – review and editing, software, formal analysis, project administration, resources, supervision, data curation.

## Conflicts of Interest

The author declares no conflicts of interest.

## Data Availability

Data sharing is not applicable to this article, as no new data were created or analyzed in this study. This is a narrative review of previously published literature.
